# Location, Location, Location: Is Membrane Partitioning Everything When It Comes to Innate Immune Activation?

**DOI:** 10.1155/2011/186093

**Published:** 2011-06-21

**Authors:** Martha Triantafilou, Philipp M. Lepper, Robin Olden, Ivo de Seabra Rodrigues Dias, Kathy Triantafilou

**Affiliations:** ^1^Department of Child Health, School of Medicine, University Hospital of Wales, Cardiff University, Cardiff CF14 4XN, UK; ^2^Infection and Immunity Group, School of Life Sciences, University of Sussex, Falmer, Brighton BN1 9QG, UK; ^3^Department of Pneumology, Bern University Hospital (Inselspital), University of Bern, 3010 Bern, Switzerland; ^4^Department of Internal Medicine V-Pneumology, Allergology and Respiratory Critical Care Medicine, University Hospital of Saarland, 66424 Homburg, Germany

## Abstract

In the last twenty years, the general view of the plasma membrane has changed from a homogeneous arrangement of lipids to a mosaic of microdomains. It is currently thought that islands of highly ordered saturated lipids and cholesterol, which are laterally mobile, exist in the plane of the plasma membrane. Lipid rafts are thought to provide a means to explain the spatial segregation of certain signalling pathways emanating from the cell surface. They seem to provide the necessary microenvironment in order for certain specialised signalling events to take place, such as the innate immune recognition. The innate immune system seems to employ germ-lined encoded receptors, called pattern recognition receptors (PRRs), in order to detect pathogens. One family of such receptors are the Toll-like receptors (TLRs), which are the central “sensing” apparatus of the innate immune system. In recent years, it has become apparent that TLRs are recruited into membrane microdomains in response to ligands. These nanoscale assemblies of sphingolipid, cholesterol, and TLRs stabilize and coalesce, forming signalling platforms, which transduce signals that lead to innate immune activation. In the current paper, we will investigate all past and current literature concerning recruitment of extracellular and intracellular TLRs into lipid rafts and how this membrane organization modulates innate immune responses.

## 1. Introduction

The general view of the cellular plasma membrane has evolved over the last twenty years from that of a homogeneous arrangement of lipids with embedded proteins towards that of a mosaic of microdomains, each having a specific protein and lipid composition [1]. Over the last couple of decades, evidence has accumulated for organisation of the plasma membrane into lipid-based microdomains or lipid rafts. A new model of membrane architecture has been suggested [2] in which the membrane is patchy with segregated cholesterol-rich portions, called lipid rafts. Lipid rafts are envisaged as islands of highly ordered saturated lipids and cholesterol that are laterally mobile in the plane of a more fluid disordered bilayer of largely unsaturated lipids [3, 4]. The hallmark of the lipid raft hypothesis are the spontaneous partitioning of lipids and proteins in discrete membrane domains, a behaviour based on their physicochemical characteristics and the possibility to recover these microdomains and their associated protein machinery as detergent-resistant entities using biochemical flotation experiments. Microdomains appear as small dynamic structures that can aggregate into larger platforms in response to various stimuli [5]. 

Currently, lipid rafts are thought to allow different protein-lipid and protein-protein interactions that temporarily compartmentalise the plasma membrane. Lipid rafts are thought to provide a means to explain the spatial segregation of certain signalling pathways emanating from the cell surface. They seem to provide the necessary microenvironment in order for certain specialised signalling events to take place. Recent studies have shown the importance of lipid raft formation in the acquired immune response. Major Histocompatibility Complex- (MHC-) restricted T-cell activation seems to be facilitated by lipid raft formation [6]. Furthermore, we have recently found that mediators of the innate immune response also concentrate in lipid rafts in order to facilitate signal transduction [7, 8], thus suggesting that both the acquired and innate immune systems utilise membrane partitioning as means of activation against invading pathogens. Crucial receptors for both innate and acquired immunity seem to oligomerize in nonrandom membrane structures, bringing together their signalling machinery. Thus accumulation of receptors within these “floating islands” on the cell membrane seems to bring together intracellularly all the adaptor molecules that are necessary for signalling. In this paper, we will investigate further the mechanisms of innate immune recognition and review past and current literature that leads us to believe that membrane partitioning and lipid rafts play a central role in innate immune activation.

## 2. The Innate Immune System

The innate immune system constitutes the most archaic part of our immune defences and has survived through years of evolution. Its function is thought to be the recognition of invading pathogens, the activation of inflammation to control the pathogen, and the subsequent activation of the acquired immune response. As part of its mechanism of activation, the innate immune system employs germ-lined encoded receptors, called pattern recognition receptors (PRRs) in order to “sense” pathogens. These PRRs recognise a restricted collection of microbial signatures, able to sense different types of microbial pathogens ranging from bacteria and viruses to fungi and spirochetes. Lipid rafts seem to be a key feature of the innate immune response, playing a crucial role in phagocytosis, receptor-receptor as well as receptor-pathogen associations as well as signal transduction. Families of PRRs, such as the Toll-like receptor family (TLR) as well as the C-type lectin family seem to localise in lipid rafts for their function thus demonstrating the importance of this membrane partitioning for the function of the innate immune response.

### 2.1. The Toll-Like Receptor Family

The TLR receptor family were the first pattern recognition receptors to be identified [9, 10]. This family of at least ten germ-line encoded receptors is able to “sense” microbial signatures and trigger activation leading to proinflammatory cytokine secretion. TLRs are expressed on immune cells and are able to distinguish a great variety of microbial ligands, such as cell wall components like lipopolysaccharide (LPS) from Gram-negative bacteria and lipoteichoic acid from Gram-positive bacteria, bacterial flagellin, CpG DNA, and viral DNA or single stranded RNA [11]. 

All identified TLRs are type I transmembrane proteins, whose intracellular domains contain regions homologous to the intracellular domains of IL-1R and are referred to as TIR domains [11]. These intracellular domains are able to trigger signalling pathways known to activate the nuclear factor kappa B (NF-*κ*B) [12, 13], which in turn leads to the secretion of proinflammatory cytokines such as TNF-*α*, IL-6, and IL-8.

TLR4 was found to recognise bacterial lipopolysaccharide (LPS) or endotoxin [14, 15]; TLR2 was found to recognise lipoteichoic acid (LTA) and peptidoglycan [16]; TLR3 was able to sense double-stranded viral RNA [17]; TLR5 was found to recognise bacterial flagellin [18], TLR7 [19] and TLR8 [20] to sense single stranded viral RNA, whereas TLR9 to recognise bacterial CpG DNA [21]. In addition, TLR2 was found to recognise different motifs including several components of Gram-positive bacteria such as peptidoglycan [22], lipoteichoic acid (LTA) [23], lipoarabinomannan [24], lipoproteins [25], and different LPS from certain Gram-negative bacteria [26], yeast [27], spirochete, and fungi [28, 29] through its unique ability to heterodimerize with TLRs 1 and 6 [30]. Studies using diacylated and triacylated lipoproteins have revealed that diacylated lipoproteins require TLR2/6 heterodimers for activation, whereas triacylated lipoproteins induce activation of the innate immune system independently of TLR6 and mainly through TLR2/TLR1 heterodimers [25, 31–35].

The membrane distribution of TLRs as well as their intracellular trafficking has only now begun to be investigated. Most TLRs (TLR1, TLR2, TLR4, TLR5, and TLR6) seem to activate cells by engaging their ligands on the cell surface, whereas TLR3, TLR7, TLR8, and TLR9 seem to trigger signalling intracellularly. These TLRs have been shown to reside in the ER and to recognise their ligands once they have been endocytosed [36, 37].

### 2.2. Innate Immune Recognition of Bacterial Endotoxin or Lipopolysaccharide

Investigations into the innate immune recognition of bacterial endotoxin led to the discovery of the TLR family. TLR4 is the most studied TLR, mainly because of its involvement with sepsis and septic shock. Sepsis is a paradoxical and complex disorder that results from an overreaction of our innate immune system to bacterial infections. The mechanisms that are designed to protect the host against infection by bacterial pathogens, either Gram-negative or Gram-positive, can lead to oversecretion of cytokines and fatal sepsis syndrome. It is now widely accepted that the overreaction of the host occurs at the level of the innate immune system and is directly linked to the recognition of bacterial cell wall components, such as lipopolysaccharide (LPS) from Gram-negative bacteria or lipoteichoic acid (LTA) from Gram-positive bacteria. Thus the recognition of bacterial products by the innate immune system under certain conditions seems to be detrimental for the host.

In the last twenty-five years, great leaps forward in our understanding of the molecular events that lead to the innate recognition of pathogens have occurred. One of the seminal discoveries has been the identification of a serum protein, lipopolysaccharide-binding protein (LBP), which binds LPS or LTA and delivers it to its cellular targets [38]. Probably the most important discovery has been that the main family of receptors employed by the innate immune system are the Toll-like receptors (TLRs).

As far as sepsis and bacterial recognition is concerned, TLR4 seems to be the central sensor of Gram-negative bacterial products [15, 39], whereas TLR2 seems to be the key receptor in activating the immune system against Gram-positive bacteria [23]. In addition to the involvement of TLRs, other accessory molecules seem to be involved. CD14 is believed to act as a transfer molecule for both Gram-negative and Gram-positive bacteria [40, 41]. In the case of LPS recognition, it has been further shown that a soluble molecule, MD-2 [42], as well as activation clusters involving several other receptors [43–45]. In the case of LTA recognition, TLR2 seems to form receptor clusters as well, comprising of at least CD14, TLR2, TLR6, and CD36 [46]. Thus we are moving away from the single-receptor model of activation, and a more complex picture is emerging. The mechanism that leads to activation seems to involve the careful interplay of several receptor molecules as well as serum proteins. Therefore such a complex orchestration of events requires a nonrandom membrane architecture specifically geared to bring receptor molecules together and trigger activation within the lipid bilayer and lipid rafts or membrane microdomains seem to provide this platform.

### 2.3. Protein-Protein Interactions in Innate Immunity: PRRs Are Part of Multicomponent Sensor Apparatuses

PRRs employed by the innate immune system have been shown to have the ability to bind and recognise conserved products of pathogens that are unique to the invading microorganisms but not to the host, it is becoming increasingly apparent that the model of a single PRR recognising foreign antigen is an oversimplified one. With the discovery of the Toll-like receptors as the main signal transducing molecules of the innate immune system, an onslaught of research has shown that PRRs are part of multicomponent sensor apparatuses.

**Figure 1:**
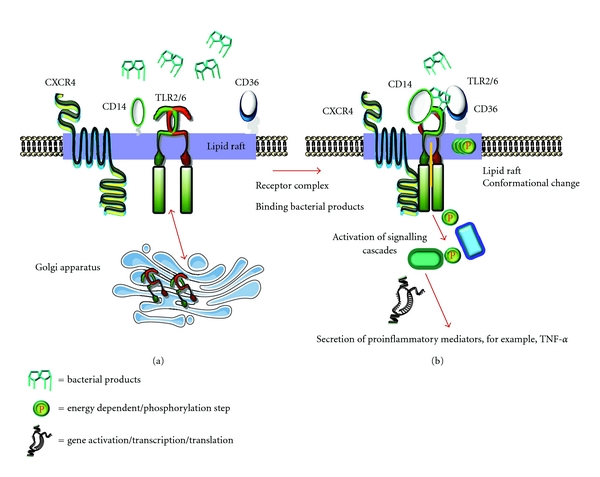
Activation of TLRs and adjuvant receptors on cell surface before and after stimulation by bacterial products. (a) TLR2 forms heterodimers with TLR1 and TLR6 on the cell surface and these heterodimer preexist and are not induced by the ligand. These heterodimers do not reside in lipid rafts before stimulation but are recruited to lipid rafts upon stimulation. This process is independent of signaling and facilitates the trafficking of TLRs from the cell surface to the Golgi. (b) TLR2/6 heterodimers are recruited within lipid rafts and associate with lipid raft-resident proteins CD14 and CD36 upon ligand stimulation. Binding of an appropriate microbial substance leads to energy-dependent clustering of heterotypic receptors and activation of intracellular signaling cascades that lead via NF-*κ*B to the production and secretion of proinflammatory cytokines.

TLRs have been shown to function as homo- or heterodimers and to even form functional interactions with non-TLR molecules. Many of these interactions are highly stable, whereas others are transient, forming dynamic associations in response to specific stimuli. Whether homotypic, heterotypic, stable, or transient, these different protein combinations generate considerable functional diversity for the innate immune system by triggering distinct signalling cascades leading to cellular activation. There are a number of examples that suggest that TLR associations are required for cellular activation. TLR4 seems to form a complex with at least two other molecules, CD14 and MD2, in order to recognise bacterial LPS [47]. In addition, it seems to associate with a Toll-like receptor homologue RP105, which acts as a negative regulator of TLR4 responses [48]. TLR2 has been found to heterodimerize with TLR1 or TLR6 for recognition of yeast components [30] and to associate with TLR1 for the recognition of bacterial lipoproteins. In addition, TLR2 has been shown to also interact with scavenger receptors in order to recognise lipoproteins [46] and most recently it was shown that TLR2 associates with CXCR4, which acts as a negative regulator of TLR2 responses [49] (Figure 1). Figure 1 depicts the possible model of TLR activation, and how TLRs and other receptors are organized in lipid rafts on the cell surface before and after stimulation. TLR2 forms heterodimers with TLR1 and TLR6. These heterodimers preexist and are not induced by the ligand (Figure 1(a)). TLR2/6 heterodimers are recruited within lipid rafts and associate with lipid raft-resident proteins CD14 and CD36 upon ligand engagement. Binding of appropriate microbial substances leads to energy-dependent clustering of heterotypic receptors and activation of intracellular signaling cascades that lead, for example, via NF-*κ*B to production and secretion of proinflammatory cytokines (Figure 1(b)).

Functional associations of TLRs with non-TLR molecules have also been demonstrated, for example, TLR2 association with dectin-1 is required for macrophage and dendritic cell activation by *β*-glucan-containing particles. More recently, functional interactions of TLR2 and CD36 have been shown to be involved in the recognition of diacylglycerides [46]. TLR4 seems to be the best example of TLRs associating with non-TLR molecules. As it has already been mentioned, TLR4 has been shown to form at least a trimolecular complex with CD14 and MD2 in order to recognise bacterial LPS [39]. The possibility that additional receptor components such as heat shock proteins [43, 50], CXCR4 [43], or CD55 [45] have been suggested to be part of this activation cluster, possibly acting as additional LPS transfer molecules. Furthermore, it has been demonstrated that different “shapes” of LPS induce the formation of different activation clusters, involving the association of TLR4 with a variety of molecules mentioned above, which seems to determine LPS responses [51]. 

Recent structural studies have shed some light onto TLR associations, supporting the hypothesis of cluster formation, since all TLRs that have been crystallised have been found to be in a dimer formation, thus the hypothesis has been put forward that dimerisation or clustering might be a common feature of the TLRs and might be essential for signalling.

Structural studies of TLRs have been an attractive area of research since structural information is crucial in understanding receptor function. In 2005, the crystal structure of TLR3 was the first one to be revealed [52]. It was surprising, that although the structure did not have a ligand, TLR3 was crystallised as a dimer. In 2007 and 2008, three structures of TLR-ligand complexes were revealed, TLR1-TLR2-lipoprotein, TLR4-MD-2-Eritoran, and TLR3- dsRNA [53–55]. The ectodomains were found to form dimers, which were strikingly similar in shape. Prior to the publication of the crystal structures, Gayand Gangloff[56] suggested a possible model of activation, where dimerization was ligand induced. These observations have suggested the hypothesis that dimerization of the ectodomains forces the intracellular TIR domains to dimerize, and this initiates signalling by recruiting the intracellular adaptor molecules, such as MyD88, MAL, TRIF, and TRAM in order to initiate signalling. The structures of the TIR domains of TLR1, TLR2, and TLR10 have been revealed [57]. Interestingly, the TIR domain of TLR10 was shown to be involved in a homodimeric interaction. However, it is not certain whether the structure seen in the crystal corresponds to a physiologically relevant dimer of TLR10 TIR domains because they have been found to exist as monomers in solution. Interestingly it has been recently suggested [58] that MyD88 interacts with IRAK4 in an 8 : 4 ratio in solution, suggesting that maybe there is higher oligomer formation. 

In order for such higher oligomers to be formed and in order to have such a well-orchestrated accumulation of receptors and signalling machinery membrane partitioning seems to be crucial for the formation of these “TLR multicomponent sensor apparatuses”.

### 2.4. TLR4 Recruitment to Membrane Microdomains upon Ligand Engagement

TLR4 was the first one to be shown to be recruited to lipid rafts upon stimulation by bacterial LPS [7]. Within these membrane microdomains it was shown that TLR4 formed clusters with non-TLR molecules that tailored the immune response against the particular pathogen [43, 59–62]. 

It was subsequently shown that this accumulation in lipid rafts also influenced its internalization and targeting. TLR4 was found to accumulate in lipid rafts, to internalize in a lipid-raft-dependent manner and to be targeted to the Golgi apparatus [63]. This intracellular targeting was shown to be independent of signalling, thus suggesting that accumulation in lipid rafts only facilitated ligand recognition and signalling that was initiated at the cell surface and not in the intracellular compartments where TLR4 was targeted to [63].

More recently it had been proposed that the molecular mechanism for signalling by the TLRs must involve a series of protein conformational changes initiated by dimerization of their extracellular domains [64]. It was suggested that this receptor-receptor association of the extracellular domains forced the association of the cytoplasmic domains as well. Motshwene et al. [58] recently proved this experimentally, demonstrating that the death domains of human MyD88, one of the adaptor proteins used by all but one of the TLRs, and IRAK4 assemble into closed complexes with stoichiometries of 7 : 4 and 8 : 4, which they called the Myddosome. The ability to form 7 : 4 or even 8 : 4 stoichiometries suggests a mechanism by which clusters of activated receptors concentrate in lipid rafts and their intracellular machinery clusters as well, forming a signalling platform that seems to be crucial for TLR activation.

### 2.5. Does Membrane Partitioning Play a Major Role in Protein Uptake and Intracellular Routing?

Cell membranes display a tremendous complexity of lipid and proteins designed to perform the functions cells require. To coordinate these functions, the membrane is able to laterally segregate its constituents. Lipid rafts were originally proposed as an explanation for a nonrandom membrane architecture and their function was originally thought to be linked with membrane trafficking. However, rafts proved to be able to influence organization of membrane receptors and bioactivity as well as membrane trafficking.

It is now emerging that this membrane partitioning might play a major role in protein uptake and intracellular routing. It is becoming more apparent that this differential sorting on the cell surface might pre-dispose the intracellular fate of a given molecule. Since the discovery of clathrin-coated pits by Roth and Porter in 1964 [65], as specialised sites for the selective recruitment of specialised molecules that are internalised into eukaryotic cells, clathrin-independent endocytic pathways have now emerged. Endocytic pathways that do not rely on the formation of clathrin coated pits include the earliest identified pathways such as phagocytosis, macropinosis, and caveolae. Lipid rafts might involved in all of these pathways. In particular for phagocytosis, it has been shown that lipid rafts play a crucial role in the phagocytic uptake of latex microspheres [66], suggesting that these specialised microdomains on the plasma membrane are necessary for endocytosis and phagocytosis.

Furthermore, caveolae which is defined as small, uncoated invaginations in the plasma membrane containing the plasma protein caveolin 1 has been shown to be able to bind cholesterol and to be resistant to detergent extraction [67], and this has led to the suggestion that caveolae might constitute a type of lipid raft [68]. Lipid rafts are increasingly becoming linked with clathrin-independent endocytosis, since nearly all molecules that are known to be internalised independently of clathrin are found in biochemically defined rafts [69]. It has been suggested that raft components might be taken up preferentially by clathrin-independent endocytosis. There are likely to be several types of clathrin-independent endocytosis. The extent to which these different pathways require lipid rafts to operate or are somehow selective for lipid rafts is currently the subject of intensive investigation. Recently, Nichols et al. have described a rapid lipid-raft-dependent targeting from the cell surface to the Golgi apparatus [70]. In addition, a new clathrin-independent mechanism has been described that can lead to delivery of receptor molecules from the plasma membrane to caveolin-1-containing endosomes, termed “caveosomes” [71]. With the emergence of these new clathrin-independent uptake mechanisms the idea that different types of endocytosis have markedly different functions is beginning to become apparent. Ultimately we have to speculate that sorting at the plasma membrane might predispose the intracellular route that a molecule might take. If that is the case, then where are the raft-associated molecules, such as TLR4, targeted to? And most importantly why?

This intracellular targeting seems to be independent of signalling. TLR2 has also been found to reside in lipid rafts after stimulation by Gram-positive bacterial products and to be similarly targeted to the Golgi apparatus [72] (Figure 1). The question that remains is whether lipid raft association is common for all TLRs expressed at the cell surface? If this is the case, do they all follow the same intracellular route? Do different signalling cascades require differential targeting of TLRs and their ligands? 

In the case of the ER-resident TLRs, very little evidence of their trafficking upon stimulation exists. To date only TLR9 has been found to translocate from the ER to lysosomes in response to its ligand, CpG DNA [73]. Based on the findings for TLR9, a hypothesis has been put forward that ER-resident TLRs might become accessible to endosomal and lysosomal compartments after the ER fuses with sites of microbial entry. If this is the case, then it would seem that ER membrane fusion might be critical for microbial recognition by ER-resident TLRs.

### 2.6. Do Lipid Rafts Control Endosomal Innate Immune Dynamics?

The regulation of endosome dynamics is crucial for fundamental cellular functions, such as nutrient intake/digestion, membrane receptor recycling/degradation, antigen presentation, cell migration, and intracellular signaling [74–76]. The system is also utilised by various pathogens to bud in and from the cell [77]. 

In addition to the function as a distribution centre, it has been proposed that the endosome system serves as an intracellular signalling station [78]. In the case of innate immunity this is certainly the case, since TLR3, TLR7, TLR8, and TLR9 all reside within the endosome waiting to capture incoming PAMPs and trigger signalling. Endosomes are pleiomorphic organelles composed of tubular elements as well as vesicular regions with a characteristic multivesicular appearance. The question that remains is whether in addition to these morphologically distinguishable regions, endosomal membranes are further subcompartmentalized into membrane lipid rafts or microdomains. 

**Figure 2:**
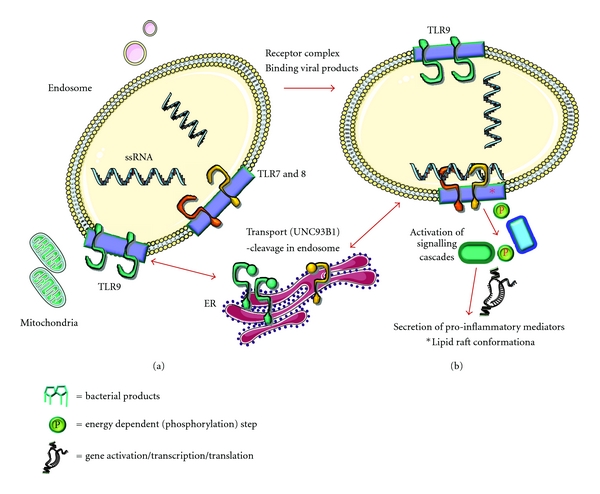
Activation of TLRs in endosomes. Various TLRs recognize microbial patterns within the endosome. TLR7 and 8 recognize ssRNA, whereas TLR9 recognises CpG DNA. As nucleic acid recognition bears a potential source for the induction of autoimmunity, TLR7 and 9 exist in a full-length and truncated version, where the ectodomain is cleaved. Only the endosomally processed forms are capable to recruit MyD88 and to induce signaling. Transport from the endoplasmatic reticulum (ER) is facilitated by UNC93B1 (a). Ligation of TLR7 by ssRNA leads to clustering within a lipid raft at the endosomal membrane and activation of intracellular signaling cascades that lead via NF-*κ*B to production and secretion of proinflammatory cytokines (b).

Lipid rafts have mostly been studied at the plasma membrane, mainly due to accessibility for microscopy and biophysical methods [79]. Characterisation of lipid rafts has also been extensively based on their resistance to detergent solubilisation, although this has inherent limitations [80], as well as fluorescent microscopy [79]. Although most studies have focused on the existence of lipid rafts on plasma membranes, many intracellular organelles appear to contain raft-like domains [81–84]. Due to its low cholesterol content, the endoplasmic reticulum was originally thought not to contain cholesterol-dependent microdomains. However, recently several studies have reported their existence [85, 86]. Raft-like domains have been described in the Golgi and trans-Golgi network [87, 88], along the endocytic pathway [84] as well as in the endosomes [89–91]. Although potential roles of lipid rafts for the outer membrane have been demonstrated, including endocytosis, exocytosis, vesicle formation, and signalling, the functions of lipid rafts in the processes of endosomal membrane dynamics are currently unknown. We can only speculate that they are contributing to similar functions. It has been suggested that protein and lipid sorting into and out of the endosomes may be controlled by endosomal membrane partitioning [89], but whether these microdomains control signalling and in particular TLR signalling has not been investigated. Since most extracellular TLRs have been shown to be recruited to lipid rafts upon ligand activation, it is safe to assume that endosomal TLRs act in a similar manner, especially since the existence of cholesterol-dependent microdomains on the endosomal membranes has been proven (Figure 2). Thus it is safe to assume that membrane partitioning control both extracellular and intracellular TLR-dependent signalling. We are proposing a model for endosomal TLR activation, where ligation of endosomal TLRs by their respective ligands can lead to clustering within lipid rafts at the endosomal membrane and activation of intracellular signaling cascades that lead via NF-*κ*B to production and secretion of proinflammatory cytokines (Figure 2(b)).

### 2.7. Existence of Other PPRs in Lipid Rafts

C-type lectin receptors (CLRs), such as Dectin 1, are a family of pattern recognition receptors which bind *β*-glucan found in the cell walls of pathogenic fungi such as *Candida albicans*. In particular Dectin 1 has been shown to mediate the phagocytosis of yeast and yeast-derived particles, such as zymosan, activating the production of inflammatory cytokines [92–94]. Interestingly, Dectin-1 possesses an immunotyrosine-activated motif (ITAM) in its cytoplasmic tail, suggesting that it is capable of mediating signalling analogous to the BCR and TCR. Since both the BCR and TCR have been found to reside in lipid rafts it was suggested that Dectin-1 might also be recruited there upon activation. A recent study has revealed that Dectin-1 and possibly other CLRs are recruited to lipid rafts upon activation and raft integrity is important for signalling [95]. Thus suggesting that recruitment to lipid rafts is a common feature for most PRRs, including TLRs and CLRs.

### 2.8. Concluding Remarks

Cell membranes are complicated in composition but precise in purpose: to selectively compartmentalize its constituents in order to coordinate cellular functions. In this way, the membrane is able to compartmentalize, segregate receptors as well as their signalling machinery and create oligomeric signalling platforms in order to transduce signals. Once the required function has subsided, these segregated islands are involved in internalization and membrane trafficking, thus bringing the whole function to a close. The innate and acquired immune systems seem to utilise this membrane partitioning for their functions. In this paper, we have extensively looked at the use of this membrane partitioning by the innate immune system and most particular by the TLRs. The molecular mechanism involved in LPS recognition and TLR signalling in general, utilises a series of protein lipid as well as protein-protein interactions. The plasma membrane seems to be heterogeneous and to coalesce to more stable membrane-ordered assemblies upon activation by ligands. This partitioning of the membrane and the assembly of more stable raft platforms in the functionalized state must be initiated by raft-resident proteins, which form protein-lipid as well as protein-protein interactions. The TLRs and other PRRs associate with the raft-resident proteins and are recruited to these “floating islands” forming higher oligomers, both extracellularly as well as inside the cell, concentrating their signalling machinery which finally leads to a functional, focused, and coordinated activation of the innate immune system.

The lifetime of these domains and the length of the response will depend on their size and factors that may stabilise or destabilise them. These factors will include not only lipid-lipid, lipid-protein and protein-protein interactions both in the plane of the membrane but also elements of the cytoskeleton, pericellular matrix adjacent to the membrane as well as transmembrane and cytoplasmic domains of the receptors involved. In the case of TLRs, the association of the TIR domains intracellularly would stabilise the ectodomains extracellularly and provide the molecular scaffold that will recruit the adaptor molecules that contribute to signalling. The challenge for the future will be to visualise the assembly and stoichiometry of these large and transient oligomeric complexes *in vivo*. Thus refining existing biophysical methods, such as single particle tracking (SPT), fluorescence correlation spectroscopy (FCS), and fluorescence resonance energy transfer (FRET) will help us reveal these dynamic nanoassemblies of sterol, sphingolipid, and proteins in living cell and provide us with the first dynamic picture of the innate immune response.
